# In silico model-guided identification of transcriptional regulator targets for efficient strain design

**DOI:** 10.1186/s12934-018-1015-7

**Published:** 2018-10-25

**Authors:** Lokanand Koduru, Meiyappan Lakshmanan, Dong-Yup Lee

**Affiliations:** 1 Department of Chemical and Biomolecular Engineering, National University of Singapore, 4 Engineering Drive 4, Singapore, 117576 Singapore; 2 Bioprocessing Technology Institute, Agency for Science, Technology and Research (A*STAR), 20 Biopolis Way, #06-01, Centros, Singapore, 138668 Singapore; 3 School of Chemical Engineering, Sungkyunkwan University, 2066, Seobu-ro, Jangan-gu, Suwon, Gyeonggi-do 16419 Republic of Korea

**Keywords:** Model-guided strain design, Genome-scale metabolic model, Constraint-based flux analysis, Transcriptional regulator, Systems biology

## Abstract

**Background:**

Cellular metabolism is tightly regulated by hard-wired multiple layers of biological processes to achieve robust and homeostatic states given the limited resources. As a result, even the most intuitive enzyme-centric metabolic engineering endeavours through the up-/down-regulation of multiple genes in biochemical pathways often deliver insignificant improvements in the product yield. In this regard, targeted engineering of transcriptional regulators (TRs) that control several metabolic functions in modular patterns is an interesting strategy. However, only a handful of in silico model-added techniques are available for identifying the TR manipulation candidates, thus limiting its strain design application.

**Results:**

We developed hierarchical-Beneficial Regulatory Targeting (h-BeReTa) which employs a genome-scale metabolic model and transcriptional regulatory network (TRN) to identify the relevant TR targets suitable for strain improvement. We then applied this method to industrially relevant metabolites and cell factory hosts, *Escherichia coli* and *Corynebacterium glutamicum*. h-BeReTa suggested several promising TR targets, many of which have been validated through literature evidences. h-BeReTa considers the hierarchy of TRs in the TRN and also accounts for alternative metabolic pathways which may divert flux away from the product while identifying suitable metabolic fluxes, thereby performing superior in terms of global TR target identification.

**Conclusions:**

In silico model-guided strain design framework, h-BeReTa, was presented for identifying transcriptional regulator targets. Its efficacy and applicability to microbial cell factories were successfully demonstrated via case studies involving two cell factory hosts, as such suggesting several intuitive targets for overproducing various value-added compounds.

**Electronic supplementary material:**

The online version of this article (10.1186/s12934-018-1015-7) contains supplementary material, which is available to authorized users.

## Background

Currently, a variety of value-added products can be newly synthesized and overproduced in microbial expression hosts at near-commercial levels through various pathway modifications such as gene up-/down-regulation and deletion in a serial and/or iterative manner [1, 2]. However, identifying such metabolic engineering targets is not trivial; more often than not, even the most intuitive enzyme manipulations may not lead to desired level of product yields due to the inherent regulation and complexity of metabolism [3]. To circumvent this issue, manipulating the transcriptional regulators (TRs), which often globally regulate the expression levels of a group of genes within a same cellular module in the form of regulons, has been considered as a promising strategy. For example, by fine tuning the expression of *FadR*, the TR regulating a number of genes in fatty acid biosynthesis including *fabA*, *fabB* and *iclR*, in *Escherichia coli*, fatty acid titres could be enhanced up to 73% of the theoretical yield which was not achieved by overexpressing any of the metabolic gene combinations [4]. Similarly, the global TR, *cra*, was targeted to channel more carbon flux via phosphoenolpyruvate carboxylation and the glyoxylate pathway in *E. coli*, thereby improving succinate yields [5]. Another recent study showed that the combinatorial overexpression of metabolic genes, *galP* and *glk*, along with a TR, *TyrR*, which represses the expression of multiple l-phenylalanine pathway genes in *E. coli*, enhanced the yield of this amino acid significantly [6]. However, despite such several success stories, one of the major challenges is to identify more efficient and reliable TR manipulation targets.

Constraint-based metabolic modeling (CBM) is a simple and widely used approach that requires only metabolic network stoichiometry and environmental constraints to describe the cellular phenotype from genotype, and thus can be readily exploited to characterize and predict cellular behaviours under perturbed conditions [7, 8]. In this regard, several algorithms based on CBM framework have been developed for finding relevant metabolic engineering targets towards the enhanced production [9–11]. While most of these algorithms can suggest various strain design strategies via gene knockout, upregulation and downregulation [9, 12], metabolite intensification/attenuation [13] and also cofactor balancing [14, 15], only a handful of them are related to TR manipulation targeting. OptORF is the first ever constraint-based method developed for TR targeting [16] using a previously developed combined metabolic/regulatory model [17] where the transcriptional-regulatory information is described via Boolean logic, i.e. ‘on’ and ‘off’ states of TR expression. A bi-level mixed-integer linear programming (MILP) based solution procedure was proposed to identify TR manipulation targets in *E. coli* for overproducing ethanol, isobutanol and 2-phenylethanol. Later, Vilaça et al. [18] used evolutionary algorithm and simulation annealing as the optimization algorithms to find TR candidates from the same combined metabolic-regulatory model. However, the use of these methods is severely limited since it assumes the transcriptional-regulatory responses to be binary which could be continuous. In order to address this critical issue, recently, Kim et al., developed Beneficial Regulator Targeting (BeReTa), on the basis of an unintegrated approach where each TR in the transcriptional regulatory network (TRN) is ranked for genetic manipulation, i.e. up-/down-regulation, based on a beneficial score [19]. A systematic procedure was proposed to combine the regulatory strength information from the TRN and the desired flux slopes that could overproduce the desired compound.

While the unintegrated approach presented in BeReTa could effectively identify several relevant TR candidates for up-/down-regulation compared to OptORF, it still suffers from certain limitations. Firstly, BeReTa does not consider the inherent hierarchical structure of TRN; unlike metabolic genes, TRs are known to operate in a regulatory cascade when certain global TRs regulate multiple downstream TRs, all of which in turn can modulate the expression of target genes [20, 21]. Here, it should be highlighted that the regulation of TR–TR-gene in TRNs are complex which at times can be circular and negate the overall effects in a counter-intuitive manner. Therefore, it is important to incorporate the hierarchical structure of TRN while identifying the TR candidates such that the engineered TR’s effect is not masked by another higher order TR. Secondly, BeReTa only takes into account the positively correlated reactions while calculating flux slopes, ignoring the reactions that are negatively correlated to the desired product which may also serve as relevant gene manipulation, i.e. down-regulation, targets. Furthermore, it does not consider the presence of equivalent competing pathways in the product synthesis that also gives rise to the same yield of product.

In this work, we propose “hierarchical-Beneficial Regulatory Targeting” (h-BeReTa), which extends the BeReTa by addressing the abovementioned shortcomings for identifying efficient TR targets. Specifically, h-BeReTa utilizes a TRN with hierarchies of TR clearly defined and a metabolic model to identify target candidates. Moreover, it also account for the negatively correlated reactions with the product flux, in addition to the positively correlated reactions because the flux through these reactions need to be minimized to improve product synthesis. Here, we first describe the methodology of h-BeReTa, and then demonstrate its applicability by identifying promising TR manipulation targets for overproducing various compounds in *E. coli* and *C. glutamicum*. Finally, we compare the resulting targets obtained from h-BeReTa to its preceding methods and discuss their performance.

## Methods

### h-BeReTa algorithm

**Fig. 1:**
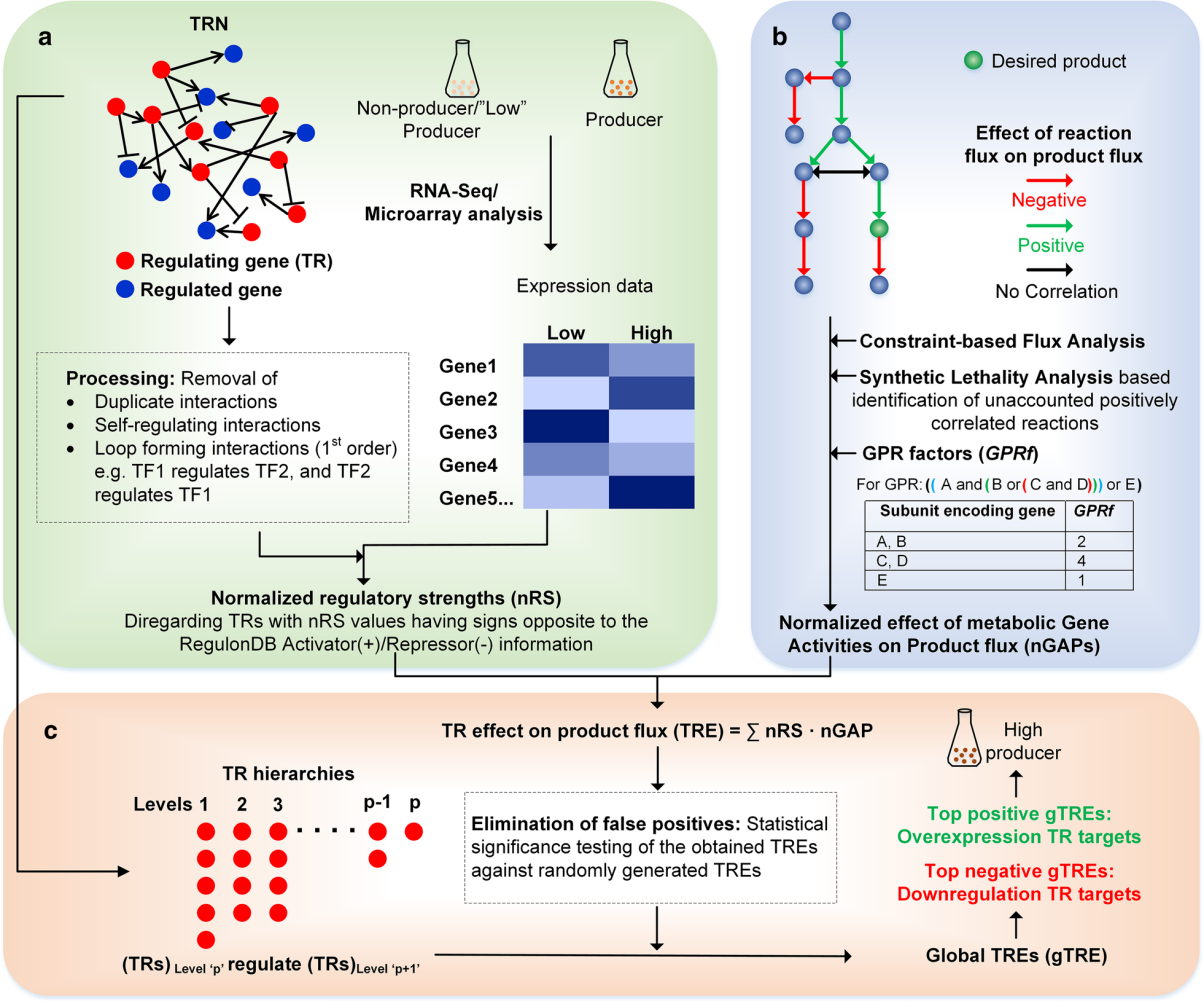
Schematic workflow of h-BeReTa. **a** Acquisition of gene-expression data for producer and non-producer, processing TRN information, determination of nRS values. **b** Constraint-based flux analysis mediated determination of nGAPs for a desired product using GEM with necessary GPRs. **c** Calculation of the effect of TRs on product flux (TREs), and therefore global TREs using nRS, nGAP values in combination with TR-hierarchy information

h-BeReTa aims to identify the relevant TR targets for up-/down-regulation to overproduce the desired product using an unintegrated approach which was previously proposed by BeReTa [19]. Initially, constraint-based flux analysis is used to identify the reactions that are both positively and negatively correlated with the desired product flux across the entire metabolic network. Subsequently, the algorithm identifies the corresponding TRs which modulate the expression of these reactions and the strength of their regulation. Finally, each TR is scored based on its regulatory strength, position in the TRN hierarchy and their association with product flux either in the positive or negative manner. The candidate genes with highest and lowest scores from the ranked list can then be chosen for their up- and down-regulations, respectively. The scoring procedure involves five key steps as summarized in Fig. 1.

### Step 1: Identification of reactions correlated with product flux (*nRAP*)

*nRAP* represents the extent of control a particular reaction flux could have on the product flux. Calculation of *nRAP* involves a constraint-based flux analysis formulation as shown below:

P1 $$\begin{aligned} & {\text{Maximize}}\;\;v_{target} \hfill \\ & {\text{Subject to}}:\;\sum _{n} S_{mn} \cdot v_{n} = 0 \hfill \\ & \alpha_{n} \le v_{n} \le \beta_{n} \hfill \\ & v_{biomass} \ge 0.5 \cdot v_{biomass}^{\text{max} } \hfill \\ & v_{r} = v_{\text{min} } + k \cdot (v_{\text{max} } - v_{\text{min} } ) \hfill \\ & \forall k = 0, \, 0.1, \, 0.2 \ldots 1\;{\text{and}}\;r \subset n \hfill \\ \end{aligned}$$

where, *v*_*t*arg*et*_ represents the product forming flux, *v*_*biomass*_ is flux through biomass forming reaction and $v_{biomass}^{\max}$ represents its maximum attainable value, *v*_min_ and *v*_max_ are minimum and maximum feasible flux values of reaction ‘*r*’ (*v*_*r*_), whose effect on *v*_*t*arg*et*_ is to be determined. *v*_min_ and *v*_max_ are determined using flux variability analysis [22]. Once the linear programming (LP) problem shown above is solved for all gene-associated reactions, denoted by ‘*r*’, the normalized Reaction Activity on Product flux (*nRAP*_*r*_) are calculated as slopes of linear plots for *v*_*t*arg*et*_ versus *k* as shown previously [19]. Here, the fractional change ‘*k*’ is chosen instead of the absolute change in the reaction flux in order to avoid unrealistically large *nRAP* values for reactions carrying small fluxes. *nRAP* can take any real value depending upon the bounds used for model simulation; reactions potentially favouring product formation take a positive value and those that have potential negative impact on product flux take a negative value. Reactions which does not influence the product flux take a value, ‘0’. Here, it should be highlighted that the solutions obtained by solving optimization problem P1 will identify only the reactions which are present in the shortest path to the product will be ranked with positive or negative *nRAP* values. However, there could be reactions that are part of alternative (or non-optimal) flux modes which could also be positively or negatively correlated to product formation (Additional file 1: Figure S1). Therefore, an additional optional step can be introduced in order to identify such reactions with appropriate *nRAP* scores (Additional file 1).

### Step 2: Identification of reactions correlated with product flux (*nRAP*)

Once the *nRAP* is calculated including the alternate pathways, the normalized effect of metabolic Gene Activities on Product flux (*nGAP*) can be computed for each gene ‘*j*’ that are associated with reaction ‘*r*’ using gene-protein-reaction (GPR) association information available in the metabolic model using below equation:

1 $$nGAP_{jr} = \frac{{nRAP_{r} }}{{GPRf_{j} }}$$

where, *GPRf*_*j*_ is GPR factor of gene ‘*j*’, which distributes the weightage of each gene ‘*j*’ associated with the reaction ‘*r*’. For example, if a reaction has GPR ((A and (B or (C and D)) or E), then, *GPR*_*f*_ of E is ‘1’ as it can form a fully functional enzyme. On the other hand, *GPR*_*f*_ of A and B is ‘2’ as each can constitute half of the multi-subunit enzyme complex; and *GPR*_*f*_ of C and D is ‘4’, as they constitute quarter each.

### Step 3: Calculation of normalized regulatory strength (nRS)

The regulatory strength (RS) represents the effect of TR expression on the expression of the downstream regulated gene. For a TR, ‘*i*’ regulating the metabolic gene ‘*j*’, the normalized regulatory strength (*nRS*_*ij*_) can be calculated as below:

2 $$nRS_{ij} = \left( {\frac{{Gene_{j,prod} - Gene_{{j,n{{\text{-}}}prod}} }}{{TR_{i,prod} - TR_{{i,n{{\text{-}}}prod}} }}} \right)*\left( {\frac{{TR_{{i,n{{\text{-}}}prod}} }}{{Gene_{{j,n{{\text{-}}}prod}} }}} \right)$$

where, *TR*_*i,prod*_ and *TR*_*i,n*-*prod*_ are the expression intensities of the TR ‘*i*’ at producer and non-producer product conditions, respectively. *Gene*_*i,prod*_ and *Gene*_*i,n*-*prod*_ are the expression intensities of the gene ‘*j*’ regulated by TR ‘*i*’ at producing and non-producing conditions. It should be noted that the “producer” and “non-producer” phenotypes can even be replaced by “slightly-better producer” and “producer” phenotypes, respectively. The absolute changes of expression intensities seldom determine the extent of the ‘overall effect’ of the change in gene expression. Hence, a normalization factor $\frac{{TR_{{i,n{{\text{-}}}prod}} }}{{Gene_{{j,n{{\text{-}}}prod}} }}$ was multiplied to the ratio of absolute changes in the gene expression levels to yield *nRS*_*ij*_. Here, the normalization is important because the TRs with low expression values are bound to receive relatively higher regulatory strengths compared to those with high expression levels, although the actual extent of regulation of their respective genes could be similar. Furthermore, the activator/repressor information obtained from RegulonDB is used to eliminate the TRs with *nRS* values having sign opposite to its known functionality.

### Step 4: TR effect on product flux (*TRE*)

The effect of each TR on product flux is calculated by combining the effect of normalized regulatory strength (*nRS*_*ij*_) and its downstream metabolic gene activities on product flux (*nGAP*):

3 $$TRE_{i} = \sum\limits_{r} {nRS_{ij} \cdot nGAP_{jr} }$$

where, *nRS*_*ij*_·*nGAP*_*jr*_ represents the effect of the TR ‘*i*’ on the product flux via the gene-associated reaction ‘*r*’.

In order to ensure that the *TRE* scores are not affected by false positives/or result of random chances, h-BeReTa calculations were performed using *nGAP* values derived from large sets (~ 1000) of *nRAP* values that are randomly generated within the observed ranges. *TRE* scores for each TR were then obtained for the 1000 randomly generated *nGAP* sets. Subsequently, the probability of the randomly generated *TRE* to fall in ± 10% range of the actual *TRE* scores of the corresponding TR is calculated. TRs with this probability less than 0.05 (5%) are considered true positives and therefore carry forwarded to Step 5.

### Step 5: Global TR effects based on hierarchies of TRN (*gTRE*)

**Fig. 2:**
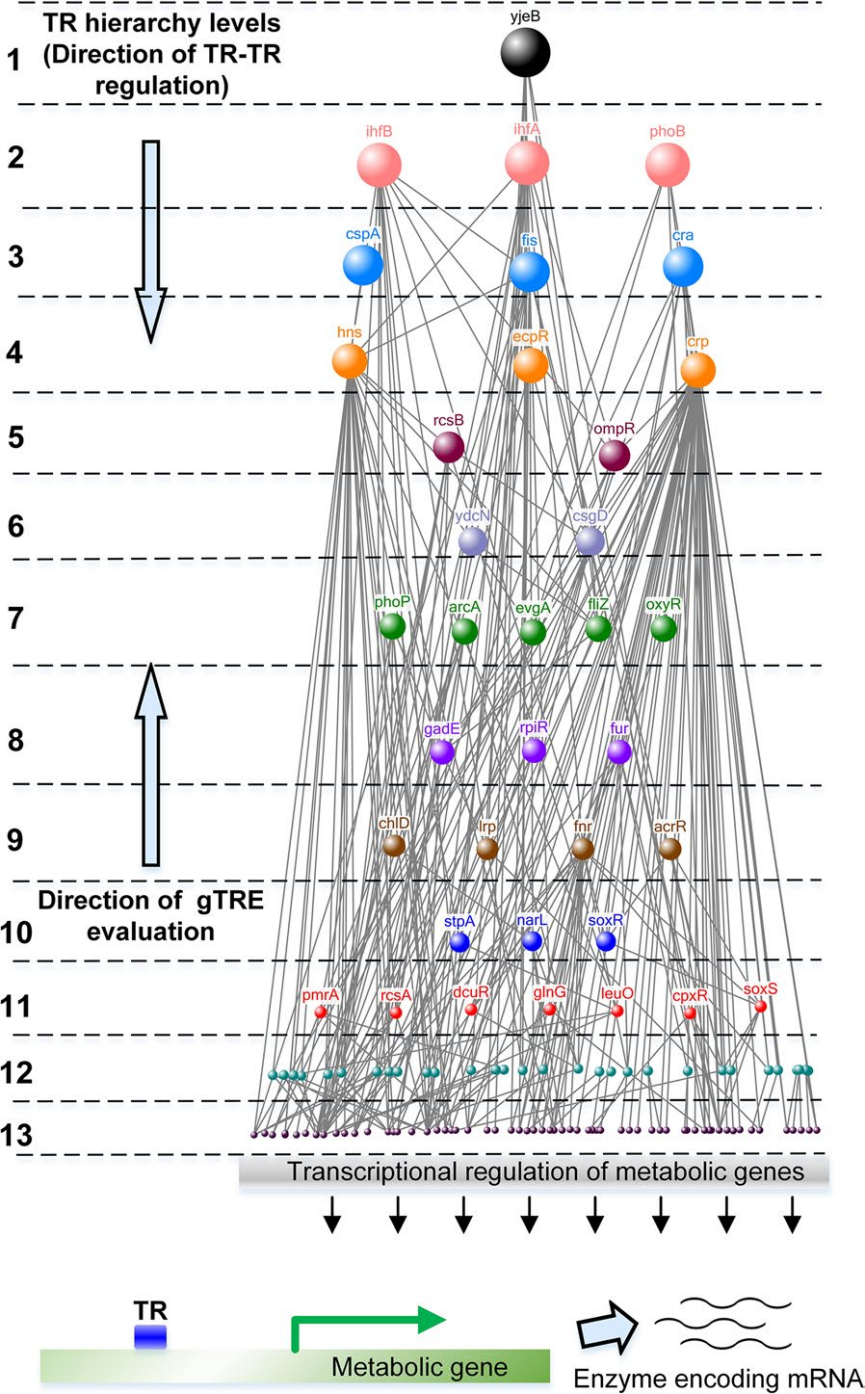
TR-hierarchy inferred from the *E. coli* regulatory network. Thirteen levels of TR–TR regulation were decoded from the TRN obtained from RegulonDB. Note that the self-regulating and loop forming TR–TR interactions are excluded from the TR-hierarchy to prevent gTREs from receiving unrealistically high values

Transcriptional regulation of metabolism includes not only the TR-metabolic gene interactions but also TR–TR interactions. Such interactions are effectively represented by TRN, which also provide information about the hierarchies of TR–TR interactions. In such hierarchies, the effect of a certain global TR on product flux (*gTRE*) would be the sum of its own effect on product flux (*TRE*) and *gTRE*s of different TRs in the immediate downstream level of the TR-hierarchy that it regulates. Hence, as depicted in Fig. 2, the calculation of *gTRE*s should be approached from the bottom-most level which only includes TRs that regulate “non-TR” genes to the top-most level which includes global TRs along with those TRs, which are not under the control of any known TRs. This effect can be calculated as follows:

4 $$gTRE_{p} = TRE_{p} + \sum\limits_{p}^{h} {nRS_{p(p + 1)} \cdot gTRE_{{p{ + 1}}} }$$

where, ‘*p*’ can take any value from 1 to ‘*h*’ and is the level immediately upstream to ‘*p *+ *1*’ in the TR-hierarchy (Fig. 2), ‘*h*’ is the total number of hierarchy levels and *nRS*_*p(p*+*1)*_ is the normalized regulatory strength of TR at level ‘*p*’ on TR at level ‘*p *+ *1*’. The summation term in the above expression exists only for those TRs that regulate other TRs and does not exist for TRs at level ‘*h*’, i.e. the last downstream level in the hierarchy. The *gTRE*s of all TRs in the TRN, thus calculated, can be used to rank them as either overexpression or downregulation target depending on whether they receive high positive or high negative values, respectively.

### In silico models and gene expression datasets

h-BeReTa require three inputs for its implementation: a genome-scale metabolic model (GEM), TRN along with the reconstructed TR-hierarchies and gene expression datasets of two reference strains.

### Genome-scale metabolic models

The *i*JO1366 [23] and *i*AF1260 [24] GEMs were used to evaluate the *nGAP* values for the *E. coli* case studies, and the *i*CW773 [25] GEM was used for *C. glutamicum* case studies. All constraint-based simulations were performed using COBRA toolbox [26], implemented in MATLAB (http://www.mathworks.com) with Gurobi5 (http://www.gurobi.com) as the optimization solver. Note that FVA was performed by employing the FastLooplessFVA function, implemented in COBRA toolbox [26] which uses a fast sparsification algorithm to efficiently eliminate the thermodynamically infeasible loops [27].

### Transcriptional regulatory networks

The TRN information of *E. coli* was downloaded from RegulonDB version 9.0 [28] including a total 4787 TR-gene interactions and 200 TRs. The TRN information of *C. glutamicum* was obtained from the Abasy Atlas database [29] which accounts for 3330 TR-gene interactions excluding self-regulators and 102 TRs. Here, it should be noted that the levels of TR regulation hierarchy were manually reconstructed from the RegulonDB TRN based on the TR–TR interaction relationships.

### Gene expression datasets

Apart from a metabolic model and TRN, h-BeReTa requires two specific gene-expression datasets relevant to the desired phenotype, i.e. “producer vs. non-producer”, for the identification of promising TR-manipulation targets. Such datasets can be obtained from the two different phases of a cell culture, e.g. growth vs. stationary phase, which shows differential transcriptional regulation. Alternatively, gene expression datasets obtained while comparing a wild-type to that of a transcriptional regulator engineered mutant can also be used for this purpose. Note that the gene expression datasets used are product-specific unlike BeReTa, which uses a general gene expression compendium for all products. The expression datasets for the case studies involving the production of tyrosine, acetate and fatty acids were downloaded using the GEO accessions provided in references cited for the respective case studies (see “Results”).

## Results

### Application of h-BeReTa to *Escherichia coli*

**Fig. 3:**
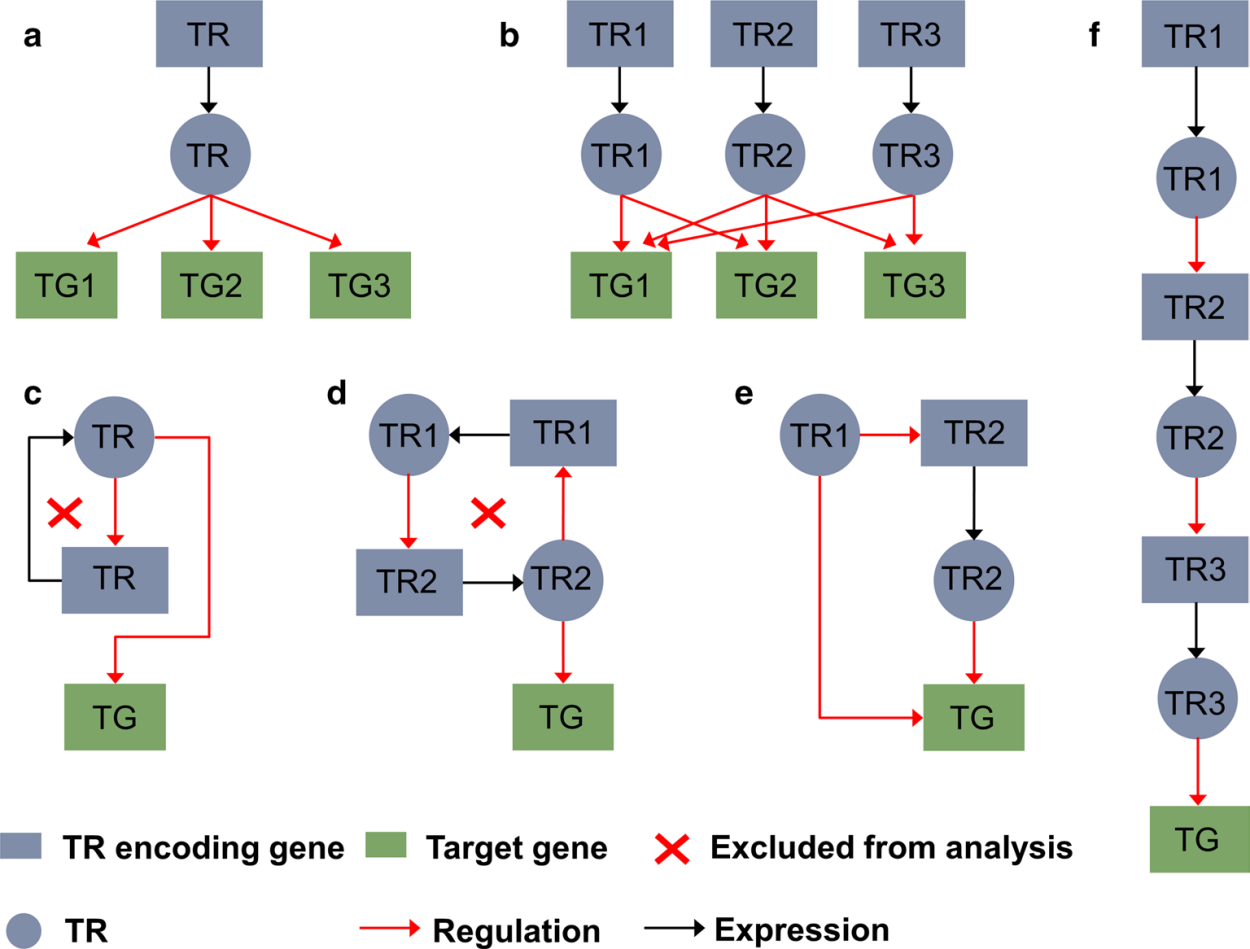
Different types of TR–TR interactions. Linear interactions represented by **a**, **b**, **e** and **f**, which result in finite gTREs were included in h-BeReTa. Interactions represented by **c** and **d**, which result in either zero or infinite gTREs, were excluded from the h-BeReTa analysis

*Escherichia coli* is one of the well-studied microbes, and a commonly used cell factory for producing various value-added compounds due to the ease of gene manipulations with abundantly available genetic engineering tools. Hence, to take advantage of such valuable resources, we used *E. coli* to demonstrate the applicability of h-BeReTa (The Matlab code for h-BeReTa is provided in https://github.com/lokanandk/h-BeReTa). We used the RegulonDB information to manually retrieve several hierarchies from the *E. coli* TRN. A total of 13 levels of TR hierarchy were obtained as compared to the previously described five levels ([20, 21]; Fig. 2). It should be noted that the self-regulating and loop forming interactions are excluded from all the levels since the net effect of a TR–TR interaction causing negative feedback will be zero and that causing positive feedback will be infinite (Fig. 3). Such exclusion prevents the assignment of potentially very high or unrealistic values to *gTRE*s. Using the reconstructed hierarchical TRN, we then applied the h-BeReTa algorithm to identify best TR manipulation targets for the overproduction of five products, including acetate, tyrosine, fatty acids, lycopene and menaquinone. All TR targets thus obtained were comprehensively mined against the published literature to retrieve possible true or false positive evidences, if any.

#### Acetate

Overflow metabolism is a cellular process observed in *E. coli* during higher aerobic growth rates, characterized by wasteful energy dissipation in the form of secretion of the fermentative byproducts such as acetate. Excess accumulation of acetate by *E. coli* causes growth inhibition, thus affecting product yields as a consequence of loss of useful carbon. Previous studies have clearly demonstrated the role of transcriptional regulation in this metabolic phenomenon. Hence, the gene expression data for the growth of *E. coli* in a chemostat [30] was retrieved from GEO, and the h-BeReTa was applied to identify the TR manipulation targets. The top overexpression and downregulation targets are presented in Table 1. The effects of several TRs on acetate production were reported in literature, importantly many of which are consistent with the top targets. It has been observed that several of these TRs control acetate flux by directly regulating enzymes involved in glycolysis, TCA cycle and glyoxylate pathway [30–32].

**Table 1:** Top-five along with additional validated (if any) transcriptional regulator targets identified by h-BeReTa for overproducing various compounds in *E. coli* and its comparison previously existing methods

Product	Nature of target	h-BeReTa	BeReTa	OptORF
Acetate	Upregulation	nac, ihfA, tyrR, rpiR, fliZ	tdcA, tdcR, **argP** [52]	–
	Downregulation	*cra* [31, 59], *crp* [60], *oxyR* [17], *fur* [61], fnr, *phoB* [62], *argP* [52]	*cra* [31, 59]	–
Tyrosine	Upregulation	*soxR* [63, 64], *soxS* [63, 64], *fadR* [65], rcsB, arcA, *cra* [53]		–
	Downregulation	acrR, adiY, gadW, flhD, argP, *tyrR* [33]	**cra** [53], *tyrR* [33], trpR, yebP	–
Fatty acids	Upregulation	fis, fnr, rpiR, yjeB, torR, *ompR* [66], *fadR* [4], **pdhR** [54]	*fadR* [4]	–
	Downregulation	fur, arcA, rcsB, oxyR, cra, *crp* [67, 68]	*pdhR* [54], cra, yijC	–
Lycopene	Upregulation	arcA, gadX, fis, ihfA, soxS	soxS	–
	Downregulation	rcsB, gadE, rcsA, ihfB, cra	cra, yebP	–
Menaquinone	Upregulation	arcA, nadI, trpR, cra, atoC		–
	Downregulation	rob, fnr, creB, tdcR, tdcA	iclR	–
Ethanol	Upregulation	fur, oxyR, glpR, envY, dnaA, **arcA** [69]	fur	–
	Downregulation	rbsE, cpxR, *ihfB* [56], *fnr* [56], *crp* [56]	*pdhR* [70], **cra** [71], gntR, kdgR	arcA-pgi, fnr-gntR-pflB-tdcE-tpiA

TRs are provided in decreasing absolute *gTRE* trends. TR targets highlighted in italic letters are true positives (TP) and those underlined are false positives (FP)

#### Tyrosine

Tyrosine has been used for a wide range of industrial and pharmaceutical applications as dietary supplements and precursors for the synthesis of benzylisoquinoline alkaloids and polyketides. Several metabolic engineering strategies have been carried out to increase the production of tyrosine in *E. coli*. Here, we apply h-BeReTa for the overproduction of tyrosine using the expression data obtained from the mutagenesis libraries of the global transcription factors rpoA and rpoD using a high tyrosine-yielding engineered parental strain [33]. The constraint-based simulations for *nGAP* determination were performed on *i*JO1366 GEM with flux through the reaction catalysed by prephenate dehydratase (*pheA*) constrained to zero, in order to mimic the metabolic state of the engineered parental strain. Interestingly, many of the TR targets identified correspond to those regulating the pool of phosphoenolpyruvate (PEP), an early precursor for tyrosine biosynthesis (Table 1). This observation clearly indicates that despite undergoing sufficient modifications in the downstream module of tyrosine biosynthesis such as the deletion of repressor gene *tyrR*, deletion of *pheA* and overexpression of feedback resistant 3-deoxy-d-arabinoheptulosonate‐7‐phosphate synthase (*aroG*^fbr^) and chorismate mutase/prephenate dehydrogenase (*tyrA*^fbr^) [33], it still has some room for further improvement. Since the original dataset reported three different regulatory modifications (*rpoA14*, *rpoA27*, and *rpoD3*) [33], we further tested the consistency of h-BeReTa predictions across all three cases. Overall, we could predict similar TR targets across all three cases using gene expression datasets which are obtained under different regulatory conditions, thus clearly indicating the robustness of h-BeReTa.

#### Fatty acids

Although bacterial hosts have been found to be a less appealing than yeasts for the industrial production of free fatty acids [34, 35], the tremendous potential of fatty acids and their derivatives for pharmaceutical and cosmetic applications and the ease to genetically manipulate have driven numerous engineering efforts in *E. coli*. The fatty acid metabolism in *E. coli* is extensively regulated at transcriptional level, and hence their overproduction would require significant interventions in the associated TRs [36]. Here, we use the expression data generated by one such study [4] to rank TR-manipulation targets for fatty acid overproduction. A synthetic reaction representing fatty acid biosynthesis was added to *i*JO1366 GEM to perform constraint-based simulations. h-BeReTa identified relevant TR overexpression and downregulation targets for fatty acid overproduction where at least three out of the four TR targets for fatty acid overproduction that were validated using experimental evidences either activate or repress fatty acid degradation (Table 1), suggesting the dominant role of β-oxidation in controlling fatty acid accumulation in *E. coli*.

#### Lycopene

Lycopene is known to be an antioxidant and a potential cancer therapeutic agent, and thus, numerous attempts have been made to produce it using engineered *E. coli* as host [37]. Initially, it has been shown that lycopene can be produced in *E. coli* via mevalonate [38] and non-mevalonate pathways [39]. However, with an increased interest for lycopene, alternative strategies are being actively sought to further enhance its yields [37]. In this regard, one of the earlier study showed that a point mutation in the global regulator, cAMP receptor protein (CRP), resulted in significant improvements to lycopene yield in *E. coli*, indicating the potential of transcriptional regulator engineering approach for lycopene production [40]. Here, we used the gene expression data obtained from the study for an *E. coli* K12 strain capable of producing lycopene and its derivative harbouring the mutant *crp* gene to predict TR engineering targets. The h-BeReTa results for TRs targets potentially improving lycopene production are listed in Table 1. The identification of *soxS*, the TR part of the *soxRS* regulon involved in relieving oxidative stress [41], as an up-regulation target is consistent with the previous observations: measurable lycopene content decreased with increasing oxidative stress [42]. Further, it should be noted that since a major portion of lycopene biosynthesis overlaps with the canonical isoprenoid biosynthesis, the TR targets obtained here can be generalized for the production of other carotenoid metabolites in *E. coli*.

#### Menaquinone (vitamin K2)

Vitamin K2 or menaquinones is a group of molecules is essential for healthy arteries and bones whose deficiency in humans could result in osteoporosis, impairment in blood coagulation and cardiovascular disease [43]. The average intake of vitamin K among the adults in the United States has been estimated to be only about 70–90% of the recommended intake value [44], emphasizing the relevance of its large-scale production to pharmaceutical and food industries. The pathway of menaquinone biosynthesis, which partially overlaps with that of aromatic amino acid and isoprenoid biosynthesis is subjected to a high level of transcriptional regulation. In this regard, we use the gene expression data obtained for the wild type *E. coli* and a mutant strain accumulating higher menaquinone pool to predict potential TR targets for vitamin K2 overproduction (Table 1). The prediction of *trpR* as an upregulation target is interesting as it represses the aromatic amino acid biosynthesis which also competes for chorismate, a common precursor for both compounds.

### Application of h-BeReTa to *Corynebacterium glutamicum*

In this work, we also applied h-BeReTa to *C. glutamicum,* an industrially important gram-positive bacterium and a representative host lesser studied compared to *E. coli*, in order to test its wider applicability. The most comprehensive TRN of *C. glutamicum* available to date [29] was used to retrieve six levels of top–down TR hierarchy (Additional file 1: Table S3). We specifically applied h-BeReTa in *C. glutamicum* to identify the TR manipulation targets for glutamate, an amino acid which it naturally produces under several conditions, and lycopene.

#### Glutamate

*Corynebacterium glutamicum* is widely used for the production of several amino acids, especially glutamate. Recently, it has been shown that *C. glutamicum* can secrete glutamate in larger amounts when exposed to the antibiotic, ciprofloxacin [45]. The gene expression data obtained in this study was therefore used here to understand the transcriptional regulation and to identify TR candidates that potentially augment the glutamate production in *C. glutamicum*. The top overexpression and downregulation TR targets are presented in Table 2 where at least one TR target each among the each category has been already reported in literature. Importantly, the prediction of *glxR* and *ramA* as overexpression targets has direct implications in decreasing glutamate yield where *glxR* is a repressor of glutamine synthase [46] and malate synthase [47], and *ramA* is a repressor of malate synthase [48], both are key enzymes in glutamate biosynthetic pathway.

**Table 2:** Top-five along with additional validated (if any) transcriptional regulator targets identified by h-BeReTa for overproducing various compounds in *C. glutamicum*

Product	Nature of target	TRs
Glutamate	Upregulation	sigA, glxR, ramA, farR, *argR* [72]
	Downregulation	cg1861, sigH, lexA, sugR, sigB, *lldR* [73]
Lycopene	Upregulation	*sigH* [49], relA, nrdR, ramB, ltbR
	Downregulation	sugR, *sigB* [49], ripA, cg2544, znr

TRs are provided in decreasing absolute *gTRE* trends. TR targets highlighted in italic letters are true positives (TP) and those underlined are false positives (FP)

#### Lycopene

Recently, it was reported that overexpression of the housekeeping sigma factor, *sigA*, resulted in more reddish coloured cells compared to the control strain of *C. glutamicum*, indicating the overproduction of lycopene [49]. Hence, we used this gene expression data obtained to characterize the transcriptional regulation of *sigA* and to suggest other TR targets to improve lycopene production even further. Interestingly, two of the TR targets identified by h-BeReTa have been validated by the same study to either increase or decrease the lycopene yields when overexpressed in *C. glutamicum* (Table 2). Here, it should be highlighted that among all targets identified, *relA* is a promising target for lycopene production, as it induces stringent response which is shown to be counteracted by one of the enzymes (4-hydroxy-3-methylbut-2-enyl diphosphate reductase or lytB or ispH) involved in the flux limiting branch point of lycopene (isoprenoid) biosynthesis [50, 51].

### Comparison of h-BeReTa with other TR-based approaches

In order to further evaluate the performance of h-BeReTa, we compared the TR targets with those obtained by BeReTa and OptORF for *E. coli* case studies. Initial comparison of the individual targets for various products from h-BeReTa and BeReTa showed a significant overlap among the resulting TRs due to the similarity in implementation (Table 1). However, h-BeReTa exclusively identified many global TRs such as *phoB*, *ihfA*, *ihfB*, *cra* and *fis* as top candidates ahead of other TRs which were commonly identified by both methods, suggesting the importance of accounting the TR-hierarchies. Moreover, the comparison also revealed a few key cases where the two methods resulted in contradicting gene manipulations for same TR targets. For example, *argP* was identified as down-regulation target for acetate production by h-BeReTa while it was predicted to be an up-regulation target by BeReTa. Similarly, h-BeReTa suggested up-regulation of *pdhR* and *cra* for improving fatty acid and tyrosine biosynthesis, respectively, whereas BeReTa predicted otherwise. To further understand why these algorithms suggested different targets for same compounds and to test their validity, we surveyed published literature. It was earlier shown that *argP* overexpression is negatively correlated with acetate production [52], confirming h-BeReTa predictions. Similarly, *cra* is shown to be a potential positive regulator of tyrosine biosynthesis through simultaneous activation of phosphoenolpyruvate synthase and repression of PTS system [53], both increasing the availability of PEP, and thus enhancing tyrosine biosynthesis. However, as *pdhR* is implicated in the repression of pyruvate dehydrogenase complex which is involved in the biosynthesis of acetyl-CoA [54], it could be a negative regulator of fatty acid biosynthesis as predicted by BeReTa. *pdhR* might therefore be a possible false positive TR target for fatty acid production predicted by h-BeReTa. We further compared h-BeReTa and BeReTa predictions through binary classification statistical tests on the basis of true positives (TP), false positives (FP) and false negatives (FN) (Additional file 1: Table S1). These results clearly demonstrate better prediction of h-BeReTa over BeReTa: it has higher sensitivity, precision and F1 score, and a low false discovery rate (Table 3). Here, it should be noted that True Negatives (TN) were not included in these tests due to the limited information available from literature sources. Finally, we also compared the results of h-BeReTa with those of OptORF for the case of ethanol production in *E. coli*. In order to obtain TR targets for ethanol overproduction, the gene expression datasets obtained from a study involving the ethanologenic strain of *E. coli* K12 grown in glucose minimal medium and a synthetic hydrolysate medium was used [55]. All three methods predicted both certain common and unique TR targets (Table 1), where only h-BeReTa was able to predict several global regulators as top targets for ethanol production in good agreement with previous reports [56]. Moreover, the TR predictions by both h-BeReTa and BeReTa also to some extent depended on the version of *E. coli* genome-scale model used to evaluate the TR targets. An overall comparison of predicted targets that are validated through experimental evidences suggested that *i*AF1260 performed better than *i*JO1366 (data not shown). While *i*JO1366 yielded unrealistic *nRAP* values for h-BeReTa possibly due to the dubious feasible flux ranges obtained using FVA, the BeReTa results using *i*JO1366 were contradictory to those of *i*AF1260, cautioning the user to ensure the reliability of the *nRAP* values or flux slopes derived from the model by evaluating them on a case-by-case basis. Moreover, h-BeReTa predictions are largely affected by the completeness of the gene expression data. When random partial gene expression datasets were used in the acetate case study, h-BeReTa predicts inferior TR targets compared to those obtained from the original datasets (Additional file 1: Table S2).

**Table 3:** Comparison of h-BeReTa and BeReTa through statistical binary classification tests

Measures	h-BeReTa	BeReTa
True positives (TP)	17	5
False positives (FP)	2	3
False negatives (FN)	10	10
FP/FN	0.2	0.3
Sensitivity or true positive rate (TPR), TP/(TP + FN)	0.63	0.33
Precision or positive predictive value (PPV), TP/(TP + FP)	0.893	0.625
False negative rate (FNR), 1-TPR	0.37	0.67
False discovery rate, 1-PPV	0.105	0.375
F1 score (0 = worst, 1 = best)	0.739	0.435

## Discussion

In this study, we introduced a new method, h-BeReTa, for identifying TRs which need to be up-/down-regulated for the overproduction of desired compounds. Unlike earlier methods, it accounts for the hierarchies of TRs in the regulatory cascade and also considers the reaction fluxes which compete with the product flux while identifying relevant TR candidates. h-BeReTa is able to identify efficient TR manipulation strategies as it is successfully demonstrated via several case studies of *E. coli* and *C. glutamicum* for overproducing various products including acetate, tyrosine, fatty acids, menaquinone, and lycopene. Here, it is important to note that the validation of the TR target predictions was only based on those examples that are available from published literature and hence, many targets remain to be validated.

As mentioned earlier, h-BeReTa utilizes an unintegrated approach which treats the cellular metabolism and regulation as two modules in the framework and then combines them systematically as previously proposed in BeReTa algorithm. However, h-BeReTa still encompasses several differences at various levels of the formulation, thereby resulting in improved performance. First, the TR hierarchy information is newly incorporated into the framework, thus identifying TR targets with higher regulatory impact on the product formation. The importance of such considerations can be perceived from h-BeReTa results which exclusively include global TRs such as *phoB*, *ihfA*, *ihfB*, *cra* and *fis* as top candidates where several of them are experimentally validated in the literature (Table 1). In addition, the TR hierarchy can provide some clues regarding the potential outcomes of global TR targeting. For example, Fig. 2 shows that *fnr* is regulated by *ihfB* (positive), *ihfA* (positive) and *fur* (negative), which occupy the upper levels of the TR hierarchy. It is furthermore clear from Table 1 that the prediction of *fnr* as an experimentally validated downregulation target for ethanol production has been consistently translated to *ihfB* and *fur* as downregulation and overexpression targets, respectively. Another important difference between the two approaches is that h-BeReTa uses a different constraint-based flux analysis formulation in which it also takes into account reactions with negative *nRAP* scores, i.e., those reactions whose fluxes compete with product formation. The inclusion of reactions with negative *nRAP* scores is important because high-value products are often secondary metabolites which the cells does not produce naturally and experiences direct competition from a large part of the fluxes in the metabolic network which are associated with biomass precursor biosynthesis. Furthermore, the accounting of negatively correlated fluxes in h-BeReTa allows it to rank the global TRs accordingly, considering that it could regulate multiple genes in other parts of the metabolic network in addition to the product flux. In contrast, since BeReTa does not consider the negatively correlated reaction fluxes there could be a bias for TRs to be identified just by considering the positive beneficial scores calculated.

Although h-BeReTa is able to identify efficient TR targets consistently, one major limitation is the inability to predict the extent of changes to product yields as a function of TR manipulation which is mainly due to the unintegrated nature of the methodology. However, the actual increase in product yield might mainly depend on several contributing factors, including the degree of correlation between the mRNA and protein levels of the TR, nature of interaction, saturation kinetics between the TR and its regulatory targets, and the intracellular metabolite concentrations, which are generally ignored in CBM approaches. Therefore, further improvements in h-BeReTa predictions could be made possible by incorporating concepts such as the metabolite dilution [57] or molecular crowding constraints [58]. Incorporation of such additional constraints into constraint-based flux analysis could potentially improve the flux predictions and therefore yield more promising TR targets. Furthermore, the use of ± 10% cut-off for assessing false positive *TRE* scores was arbitrary and can be subjected to scrutiny. However, with this cut-off range we observed a minimal rejection of true positive (literature validated) TR targets. Additionally, using more than one set of transcriptomic data representing the desired phenotype, i.e. producer and non-producer, to calculate the regulatory strength (*nRS*) values may increase the accuracy of TR candidate predictions. Alternatively, if no relevant datasets could be found for the desired phenotype, a general gene expression compendium can be used as it is in BeReTa.

Despite its limitations and scope for further improvements, the agreement of h-BeReTa predictions with experimental evidences from literature was substantial. Although the gene expression datasets used in this study for various case studies correspond to exponentially growing cells cultures, the method can also be readily extended to those of stationary phase cultures, provided an appropriate objective function is employed during the computation of *nRAP* scores. We believe that the less-stringent resource requirements and the computationally less-intensive methodology make h-BeReTa to be more readily employed in comparison to the existing methods for identifying non-intuitive TR targets, thereby advancing metabolic engineering applications.

## Additional file

**Additional file 1. Table S1.** False-negative TRs for the E. coli case studies from literature evidences. **Figure S1.** Simplified toy network showing alternate routes positively correlated to product formation. **Table S2.** Randomly selected Partial (Half) gene expression datasets for acetate case study. **Table S3.** TR-hierarchy of *C. glutamicum*.
