# Supplementary material for: In silico model-guided identification of transcriptional regulator targets for efficient strain design
Source: Microb Cell Fact. 2018 Oct 25;17:167. doi: 10.1186/s12934-018-1015-7 (PMC6201637; doi:10.1186/s12934-018-1015-7)
Supplement: Supplementary file 1 — Additional file 1. Table S1. False-negative TRs for the E. coli case studies from literature evidences. Figure S1. Simplified toy network showing alternate routes positively correlated to product formation. Table S2. Randomly selected Partial (Half) gene expression datasets for acetate case study. Table S3. TR-hierarchy of C. glutamicum. [file 12934_2018_1015_MOESM1_ESM.docx]

**Manuscript Title:** *In silico* model-guided identification of transcriptional regulator targets for efficient strain design

**Authors:** Lokanand Koduru, Meiyappan Lakshmanan, Dong-Yup Lee

**Table S1. False negatives from literature along with their references**

|  | **False negatives in *E. coli*** | **References** |
| --- | --- | --- |
| **Acetate** | fadR (Overexpression target) | [1,2] |
|  | arcA (Overexpression target) | [3,4] |
|  | iclR (Overexpression target) | [3,4] |
| **Tyrosine** | csrA (Downregulation target) | [5,6] |
|  | csrB (Overexpression target) | [7] |
|  | evgA (Overexpression target) | [8] |
| **Fatty acids** | No false negatives were found |  |
| **Lycopene** | appY (Overexpression target) | [9] |
|  | clr (Overexpression target) | [9] |
|  | yjiD (Overexpression target) | [10] |
| **Ethanol** | ihfA (Overexpression target) | [11] |
| **Menaquinone** | No false negatives were found |  |

**Table S2. Randomly selected Partial (Half) gene expression datasets for acetate case study. Bolded TRs are validated using literature evidences. Bolded-Red ones are False Positives.**

| **Targets** | **Rand set 1** | **Rand set 2** | **Rand set 3** | **Rand set 4** | **Rand set 5** | **Rand set 6** | **Rand set 7** | **Rand set 8** | **Rand set 9** | **Rand set 10** |
| --- | --- | --- | --- | --- | --- | --- | --- | --- | --- | --- |
| **Overexpression** | fnr | fnr | hdfR | gatR | leuO | pdhR | fis | narL | pdhR | cdaR |
|  | ybbI | ybbI | gatR | prpR | fis | cdaR | sgrR | cdaR | **crp** | fnr |
|  | pdhR | pdhR | gclR | leuO | cusR | sgrR | hupB | **phoB** | hns | **cra** |
|  | **crp** | **crp** | rcsB | narL | **fur** | fis | **fur** | AscG | ihfB | **arcA** |
|  | **fur** | **fur** | sgrR | AscG | **cra** | **crp** | **oxyR** | prpR | lrp | ybiQ |
| **Downregulation** | **phoB** | mraZ | cdaR | cusR | mraZ | hupB | mraZ | ybiQ | bolA | **oxyR** |
|  | **arcA** | cdaR | hns | **fur** | cdaR | betI | **crp** | fnr | mraZ | **fur** |
|  | prpR | **phoB** | bolA | chlD | **crp** | ribX | **cra** | **cra** | rpiR | betI |
|  | dpiA | gclR | **crp** | **phoB** | pdhR | nagC | **phoB** | cusR | cdaR | yedW |
|  | caiF | sgrR | leuO | ihfB | nagC | leuO | prpR | sgrR | fnr | nagC |
| **F1 Score** | **0.2** | **0.2** | **0.2** | **0.364** | **0.2** | **0** | **0.5** | **0.2** | **0** | **0.5** |

**Accounting for alternate positively correlated reactions**

In constraint-based model simulations, it is a general consensus that the reactions essential for the production of a desired metabolite are positively correlated to it. These reactions can be readily identified by the formulation described earlier. Moreover, each essential reaction may further be replaced by an equivalent set of previously unidentified reactions, part of alternate routes, which together form an essential reaction group. For instance, from the toy network depicted in the **Figure S1**, D-I, C-H-K, and B-G-J-L can be inferred as essential reaction groups. Hence, flux through such reaction groups will also be positively correlated to product formation, and can be identified using synthetic lethality analysis by setting the objective to maximization of product formation. Here, we use Fast-SL [12], an algorithm capable of performing synthetic lethality analysis in an efficient manner, to identify those reactions that form a lethality order of up to ‘4’. To achieve this, Fast-SL implementation required a slight modification, as the original implementation could only identify reaction lethality up to order ‘3’ (Supplementary information). The resultant synthetic lethal reaction groups were assigned *nRAP* values as shown below

$$nRAP=\frac{nRAP_{EssRxns}}{2^{m}}$$

Where, $nRAP_{EssRxns}$ correspond to *nRAP* values of the 1^st^ order lethal reactions (essential reactions) obtained from the original constraint-based formulation (equations 1-5) and ‘m’ is the order of reaction lethality. 2^m^ is a penalty factor that accounts for the distance from the optimal flux distribution. The index ‘2’ is used in the penalty factor because the modified FastSL implemented as a part of *h*-BeReTa involves an iterative replacement of one of the reactions from each previously identified lower order (m) synthetic lethal pairs with two new reactions which collectively form a higher order (m+1) synthetic lethal pair.


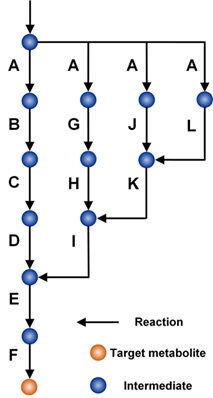


**Figure S1. Simplified toy network showing alternate routes positively correlated to product formation.** As depicted, reaction A lies at the top of the network, the product of which is involved in alternate routes for the biosynthesis of the target metabolite. Clearly, as E and F are essential for target metabolite biosynthesis, they are considered positively correlated to it and hence take positive nRAP scores. However, as the constraint-based formulation shown in Step-2, section 2.1 fails to identify A, B, C and D as positively correlated, due to the presence of alternate routes comprising of G, H, I, J, K and L, modified version of Fast-SL, an efficient method used for synthetic lethality analysis, with target metabolite maximization objective can be employed to identify the combination of reactions whose deletion diminishes product synthesis, D-I, C-H-K and B-G-J-L. Whereas, D-I form 2nd order lethal pair, H-K and J-L form 3rd and 4th order lethal pairs, respectively. The evaluation of nRAP values of each reaction identified through this analysis depend on their respective order of lethality (see methods).

**Table S3.** **TR-hierarchy of *C. glutamicum***

| **TR Level 1** | cg2092 |
| --- | --- |
| **TR Level 2** | cg3224, cg1861, cg3202, cg3253, cg2115, cg2103, cg2109, cg2544, cg2500, cg2624, cg0146 |
| **TR Level 3** | cg0350, cg0876, cg0156, cg2114, cg1120 |
| **TR Level 4** | cg0878, cg3097, cg3420, cg2831 |
| **TR Level 5** | cg0337, cg0444 |
| **TR Level 6** | cg1552, cg1084, cg2737, cg1410, cg1340, cg1738, cg0343, cg2615, cg3239, cg2502, cg0112, cg0800, cg2268, cg2888, cg1324, cg1218, cg1211, cg1032, cg0741, cg1831, cg2894, cg0309, cg2309, cg2112, cg1585, cg0646, cg0313, cg0862, cg2641, cg0317, cg2936, cg1308, cg0012, cg0019, cg2783, cg0500, cg1648, cg0463, cg3261, cg3082, cg3315, cg2910, cg0695, cg1486, cg2627, cg0993, cg2200, cg2118, cg1271, cg2516, cg1053, cg0051, cg3285, cg0454, cg0986, cg0527, cg2102, cg2965, cg1817, cg3352, cg0196, cg1704, cg3247, cg0090, cg2766, cg3388, cg1846, cg2942, cg1935, cg0979, cg3373, cg0371, cg2152, cg1547, cg1425, cg1765, cg0702, cg0565, cg0897 |

**Supplementary References**

1. Farmer WR, Liao JC. Reduction of aerobic acetate production by Escherichia coli. Appl Environ Microbiol. 1997;63:3205–10.

2. Maloy SR, Nunn WD. Role of gene fadR in Escherichia coli acetate metabolism. J Bacteriol. 1981;148:83–90.

3. Waegeman H, Beauprez J, Moens H, Maertens J, De Mey M, Foulquié-Moreno MR, et al. Effect of iclR and arcA knockouts on biomass formation and metabolic fluxes in Escherichia coli K12 and its implications on understanding the metabolism of Escherichia coli BL21 (DE3). BMC Microbiol. 2011;11.

4. Waegeman H, De Lausnay S, Beauprez J, Maertens J, De Mey M, Soetaert W. Increasing recombinant protein production in Escherichia coli K12 through metabolic engineering. N Biotechnol. 2013;30:255–61.

5. Harker M, Bramley PM. Expression of prokaryotic 1-deoxy-d-xylulose-5-phosphatases in *Escherichia coli* increases carotenoid and ubiquinone biosynthesis. FEBS Lett. 1999;448:115–9.

6. Sabnis N a, Yang H, Romeo T. Pleiotropic regulation of central carbohydrate metabolism in Escherichia coli via the gene csrA. J Biol Chem [Internet]. 1995;270:29096–104. Available from: http://www.ncbi.nlm.nih.gov/pubmed/7493933

7. Yakandawala N, Romeo T, Friesen AD, Madhyastha S. Metabolic engineering of Escherichia coli to enhance phenylalanine production. Appl Microbiol Biotechnol. 2008;78:283–91.

8. Santos CNS, Xiao W, Stephanopoulos G. Rational, combinatorial, and genomic approaches for engineering L-tyrosine production in Escherichia coli. Proc Natl Acad Sci [Internet]. 2012;109:13538–43. Available from: http://www.pnas.org/cgi/doi/10.1073/pnas.1206346109

9. Kang MJ, Lee YM, Yoon SH, Kim JH, Ock SW, Jung KH, et al. Identification of genes affecting lycopene accumulation in Escherichia coli using a shot-gun method. Biotechnol Bioeng. 2005;91:636–42.

10. Jin YS, Stephanopoulos G. Multi-dimensional gene target search for improving lycopene biosynthesis in Escherichia coli. Metab Eng. 2007;9:337–47.

11. Kargeti M, Venkatesh K V. The effect of global transcriptional regulators on the anaerobic fermentative metabolism of: Escherichia coli. Mol Biosyst. 2017;13:1388–98.

12. Pratapa A, Balachandran S, Raman K. Fast-SL: An efficient algorithm to identify synthetic lethal sets in metabolic networks. Bioinformatics. 2015;31:3299–305.
